# Transverse Tibial Bone Transport Enhances Distraction Osteogenesis and Vascularization in the Treatment of Diabetic Foot

**DOI:** 10.1111/os.13416

**Published:** 2022-08-10

**Authors:** Shuanji Ou, Changpeng Xu, Yang Yang, Ya Chen, Wenjun Li, Hanyu Lu, Guitao Li, Hongtao Sun, Yong Qi

**Affiliations:** ^1^ Department of Orthopaedics Guangdong Second Provincial General Hospital Guangzhou China

**Keywords:** angiogenesis, diabetic foot ulcer, distraction osteogenesis, transverse tibial bone transport

## Abstract

**Objective:**

To investigate the effect of transverse tibial bone transport on the treatment of Wagner Stage 4 diabetic foot.

**Methods:**

From January 2017 to October 2019, a total of 19 patients with Wagner Stage 4 diabetic foot ulcers were recruited. All patients were treated with transverse tibial bone transport. A detailed follow‐up was carried out at 1 week, 1 month, 3 months, 6 months, and 1 year after surgery. The wound healing rate and the limb salvage rate at 1 year after the surgery were evaluated. Preoperative and 3‐month postoperative digital subtraction angiography (DSA) were obtained. The level of vascular endothelial growth factor (VEGF), basic fibroblast growth factor (bFGF), epidermal growth factor (EGF) and platelet‐derived growth factor (PDGF) before surgery and on 1st, 4th, 11th, 18th, 28^th^, and 35th days after surgery were measured. Operation time, intraoperative blood loss, postoperative complications, visual analog scale (VAS) pain score, skin temperature, Semmes‐weinstein monofilament (SWM), and ankle brachial index (ABI) were also assessed.

**Results:**

The wound healing rate and the limb salvage rate were both 94.74% in the patients at 1 year after the surgery. DSA showed the thickening of the calf and foot arteries, clear visualization, and a rich vascular network. The levels of VEGF, bFGF, and PDGF on the 11th, 18th, 28^th^, and 35th days after surgery were significantly higher than those before surgery (*p* < 0.05). The EGF level on the 18th, 28th, and 35th days after surgery was significantly higher than that before surgery (*p* < 0.05). Superficial wound complications occurred in one patient during the hospitalization. There was no movement area infection, skin flap necrosis, tibial fracture, loosening of the external fixator, or rupture in study.

**Conclusion:**

Transverse tibial bone transport can improve the blood circulation of the affected limbs, promote the healing of diabetic foot wounds, and reduce the amputation rate of the affected limbs. Transverse tibial bone transport can promote the healing of Wagner Stage 4 diabetic foot.

## Graphical abstract

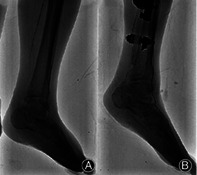

## Introduction

Diabetic foot syndrome refers to foot infection, ulceration, and deep tissue destruction caused by lower extremity neuropathy and vascular disease in diabetic patients^1^. Ulcer and amputation are the usually final outcomes of diabetic foot. According to the 2015 epidemiological data from the International Diabetes Federation for diabetic foot, 910 to 26.1 million people worldwide suffer from diabetic foot syndrome each year^1^. Between 19% to 34% of diabetic patients may have diabetic foot ulcers in their lifetime^2^. Ulceration in diabetic foot is considered as a high‐risk factor for amputation^3^, ^4^. The Wagner system for diabetic foot was widely used to assess ulcer depth and the presence of osteomyelitis or gangrene, and ranges from Grade 0–Grade 5. For Grade 4, there is partial foot gangrene in diabetic foot^5^. For the treatment of Wagner Stage four, negative‐pressure wound therapy, interventional therapy, antibiotics, vascular reconstruction, and amputation can be chosen^6^, ^7^, ^8^, ^9^, ^10^, ^11^.

However, there is still a lack of effective treatment for diabetic foot ulcer, and limb salvage therapy is a worldwide problem. The refractory condition of diabetic foot is that the peripheral nerves and peripheral blood vessels of the lower limbs are damaged to varying degrees, and the sustained high blood sugar level can accelerate blood vessel damage. The reconstruction of foot blood circulation is the premise of the treatment of diabetic foot ulcer at Wagner Grade 4. Based on the Ilizarov tension‐stress rule, the distraction osteogenesis technique promotes the growth and regeneration of osseous and soft tissues^12^, ^13^. The Ilizarov technique creates transverse distraction of the tibia by dispersing a rectangular bone fragment separated from the diaphysis without altering limb length, increasing the blood supply to the entire limb. Patients with chronic ischemia have been successfully treated with the transverse distraction technique based on histogenesis^14^. Sustained slow traction can stimulate cell proliferation, biosynthetic functions and activate new and old tissue metabolism. By giving the skeleton a suitable stretch stress, it can mobilize the potential for natural repair of the tissue, which enables the bone and its attached fascia, muscle, nerve, and blood vessels to grow synchronously^15^. It is reported that through Ilizarov transverse tibial bone transport and microcirculation reconstruction technique, tibiae are formed into movable bone flaps to be transversely transported correspondingly, which repeatedly stimulates the regeneration of tibial bone marrows, promotes neovascularization and bone tissue formation, achieves the reconstruction of peripheral blood circulation so as to improve limb blood supply, fundamentally eradicates the source of ischemic diseases in lower limbs, and promotes blood circulation, thus playing a role in clinical treatment^16^. Therefore, Ilizarov transverse tibial bone transport may be an alternative strategy for the treatment of diabetic foot. In this study, we attempted to (i) investigate the effect of transverse tibial bone transport on the treatment of diabetic foot and (ii) evaluate the vascularization and complications after transverse tibial bone transport.

## Methods

### Inclusion and Exclusion Criteria

The patients were selected based on the following inclusion criteria: (i) patients suffered from diabetes mellitus; (ii) Wagner Stage 4. All included patients were treated with transverse tibial bone transport. The exclusion criteria included: (i) complete blockage of the popliteal artery; (ii) inability to tolerate surgical treatment; (iii) poor heart and lung function, and severely poor liver and kidney function.

The Wagner system for diabetic foot was widely used to assess ulcer depth and the presence of osteomyelitis or gangrene, including Grade 0–Grade 5. For Grade 4, there is partial foot gangrene in diabetic foot.

### Study Design

The study was a single arm, assessor‐blinded, observational trial, which was performed in our hospital with a 12‐month follow‐up. All procedures were performed by a senior orthopaedic surgeon. The operating surgeon never conducted the clinical follow‐up. All clinical and experimental outcomes were assessed by a well‐trained physician who was blinded to this study. The written informed consent has been obtained from all the patients. This study was approved by the institutional review board of Guangdong Second Provincial General Hospital (GD2H‐IRB‐SC‐KY‐07‐01.0).

### Anesthesia and Position

All procedures were performed under nerve block anesthesia. The patients were placed in the supine position, and a tourniquet was not used on the affected limbs.

### Wound Debridement

Debridement is a salient component of facilitators to wound healing. These facilitators include good nutrition, wound protection, a moist wound environment, adequate oxygen supply, appropriate bioburden, and amelioration of the cause of the wound if possible. Topical cleansing products include antiseptics, antibiotics, detergents, surfactants, saline, and water. According to the wound conditions, in the first stage, all patients underwent wound debridement and the removal of necrotic tissue and purulent fluid. The surgeon paid attention to the exploration of the deep plantar layer and ensured unobstructed drainage after surgery, while bacterial culture of necrotic tissue was performed at this time. After the wound infection was basically controlled, transverse tibial bone transport was performed in the second stage.

### Transverse Tibial Bone Transport

**Fig. 1:**
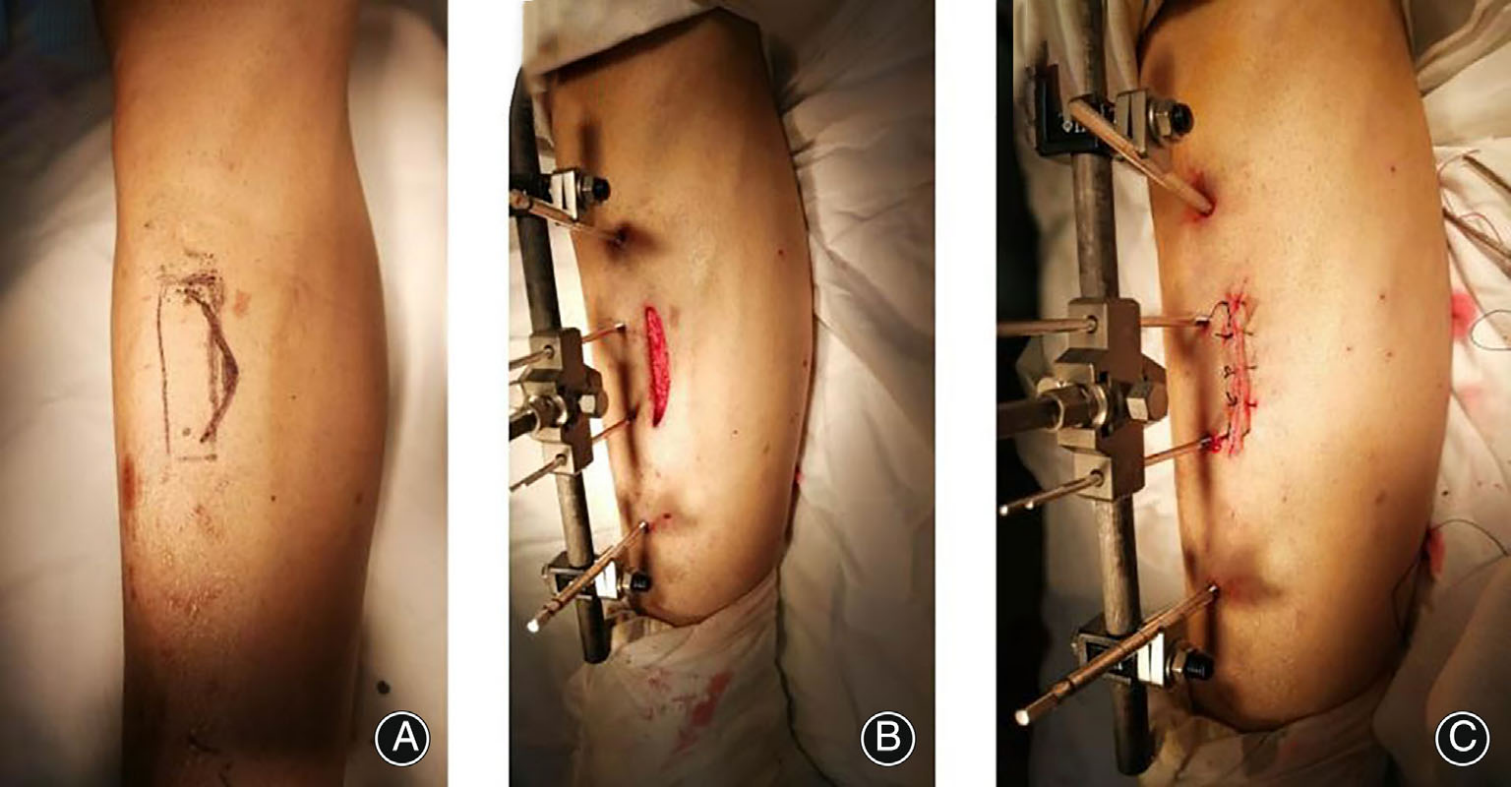
Intraoperative photograph. (A) the surgical position; (B) the osteotomy position; (C) the external mounting position

The anterior medial part of the middle and lower leg was used as the transverse bone movement area of the tibia. The tibial window was labeled with a size of 7 cm × 1.8 cm × 1.5 cm (Figure 1A). An arc incision of approximately 6 cm to 8 cm in the middle and lower part of the lower leg was made (Figure 1B). A direct incision of the skin and subcutaneous tissue to the periosteum was made, followed by a longitudinal incision of the tibial periosteum open to both sides, with the surgeon paying attention to protecting the integrity of the periosteum, exposing the tibial window area, and drilling holes one by one around the bone window. Then, the osteotome was used along the drill line to cut the bone cortex carefully, avoiding injury to the endomedullary periosteum, ensuring that the bone window formed a movable bone flap. Using a fixed needle, the surgeon drilled into the distal and proximal tibia and drilled transverse traction pins into the bone window. The external fixator (Figure 1C) was used to control the direction of transverse bone movement. The nut can control the lateral movement of the tibial osteotomy. The counterclockwise rotation can lift the tibial osteotomy (1 mm per day, 0.25 mm per time, four times) and the clockwise rotation can press back the tibial osteotomy (1 mm per day, 0.25 mm per time, four times). The wound was rinsed, bleeding was stopped, and the periosteum was sewn layer by layer, followed by the subcutaneous tissue and skin. The incision was bandaged, and the operation was completed.

### Perioperative Management

Blood sugar, blood lipids, blood pressure, and infection in correcting anemia and hypoproteinemia were strictly controlled. Appropriate elevation of the affected limb reduces swelling. The calf was first cleaned to disinfect the wound, then foot wound dressing was changed, epidermal growth factor was applied, and the external fixation nail was washed with ethanol. On the second day after surgery, an X‐ray image of the lateral side of the affected tibia was performed. After 4 days, the transversely transported tibial bone was moved laterally four times on the 5th day after the operation (1 mm/day, 0.25 mm each time). It is necessary to pay close attention to the tension and blood supply of the flap in the movement area. Once ischemic change occurs in the flap, movement should be stopped. The osteotomy block should be pressed back to avoid the necrosis of the flap and should be removed from the affected side after 2 weeks of movement. An X‐ray image of the tibia was taken in a lateral position. At this time, movement was stopped for 3 days, and then the lateral bone was moved back 1 mm/day, 0.25 mm each time, four times in total. Over 2 weeks, the tibial bone window was reset, and an X‐ray image was taken. An X‐ray image of the lateral side of the affected tibia was taken again 6–8 weeks later to examine the healing of the fracture line around the transversely transported tibial bone. If no abnormal findings were observed, the external fixator was removed. No special offloading measurements were used, and all patients wore regular shoes after ulcer healing.

### Clinical and Radiographic Assessment

A detailed follow‐up was carried out preoperatively at 1 week, 1 month, 3 months, 6 months, and 1 year after the surgery. The wound healing rate was calculated, and the rate of limb salvage was determined at 1 year after surgery. The effect of the treatment was evaluated by the wound healing of the diabetic foot and the limb salvage rate 1 year later. In addition, operation time, intraoperative blood loss, postoperative complications, visual analog scale (VAS) pain score, skin temperature, and ankle brachial index (ABI), Semmes‐weinstein monofilament (SWM) test were used to assess clinical consequences. The preoperative, immediate postoperative, and 2‐week and 3‐month postoperative standard anteroposterior and lateral radiographs of the tibia were obtained for all patients. Preoperative and 3‐month postoperative DSA on the vascular condition were obtained.

### Experimental Evaluation

Serum samples were collected before surgery and on the 1st, 4th, 11th, 18th, 28th, and 35th days after surgery. Serum was collected posttreatment and stored at −80°C until use. Vascular endothelial growth factor (VEGF), basic fibroblast growth factor (bFGF), epidermal growth factor (EGF), and platelet‐derived growth factor (PDGF) enzyme immunoassay kits (Hangzhou Lianke Biotechnology Co. Ltd., Hangzhou, China) were used to determine VEGF, bFGF, EGF, and PDGF levels according to the manufacturer's instructions.

### Statistical Analysis

The statistical analyses were conducted with SPSS 19.0 software (SPSS Inc., Chicago, IL). The quantitative data were expressed as the mean ± the standard deviation. An independent samples t‐test was used for statistical comparisons before and after surgery. *p* < 0.05 was considered as statistically significant difference.

## Results

### General Results

From January 2017 to October 2019, a total of 30 patients (30 feet) were screened. Finally, 19 feet (63.34%) in 19 patients meet the inclusion criteria and underwent transverse tibial bone transport. Ten (52.63%) patients had left foot tibial bone transport and nine (47.37%) patients had right foot tibial bone transport. One patient was lost to follow‐up because of changing their telephone number. Thus, complete clinical outcomes and radiographic data were available for 18 (94.74%) feet. The mean age of the patients was 67.00 ± 11.93 years old (Table 1). There were nine male and 10 female patients. All the patients had no lower extremity arterial disease.

**TABLE 1:** Demographics of the patients

Parameters	Value of number
Number of patients	19
Diabetes mellitus, type 2	19
Hypertension	10
Number of foots	19
Mean (SD; range) age (y) at operation	67.00 (11.93; 48–89)
Gender (female/male)	10/9
Mean (SD; range) BMI (kg/m^2^)	21.69 (2.49; 16.89–25.39)
Left foot	12
Right foot	7

Abbreviations: BMI, body mass index; SD, standard deviation.

### Surgical and Postoperative Data

The follow‐up time was 14.00 ± 1.00 months in this study. The surgery time was 58.32 ± 13.31 mins, while the blood loss during the surgery was 54.74 ± 20.65 ml. The foot wound healing time was 30.79 ± 9.70 days. The wound healing rate and the limb salvage rate were both 94.74% in the patients at 1 year after the surgery (Table 2).

**TABLE 2:** Surgical and postoperative data

Parameters	Value of number
Mean (SD; range) follow‐up time (mon)	14.00 (1.00; 12.00–15.00)
Mean (SD; range) surgery time (min)	58.32 (13.31; 40.00–90.00)
Mean (SD; range) operative blood loss (ml)	54.74 (20.65; 30.00–100.00)
Mean (SD; range) wound healing time (d)	30.79 (9.70; 21.00–60.00)
Wound healing rate (%)	18 (94.74%)
Limb salvage rate (%)	18 (94.74%)

Abbreviation: SD, standard deviation.

### Wound Healing

**Fig. 2:**
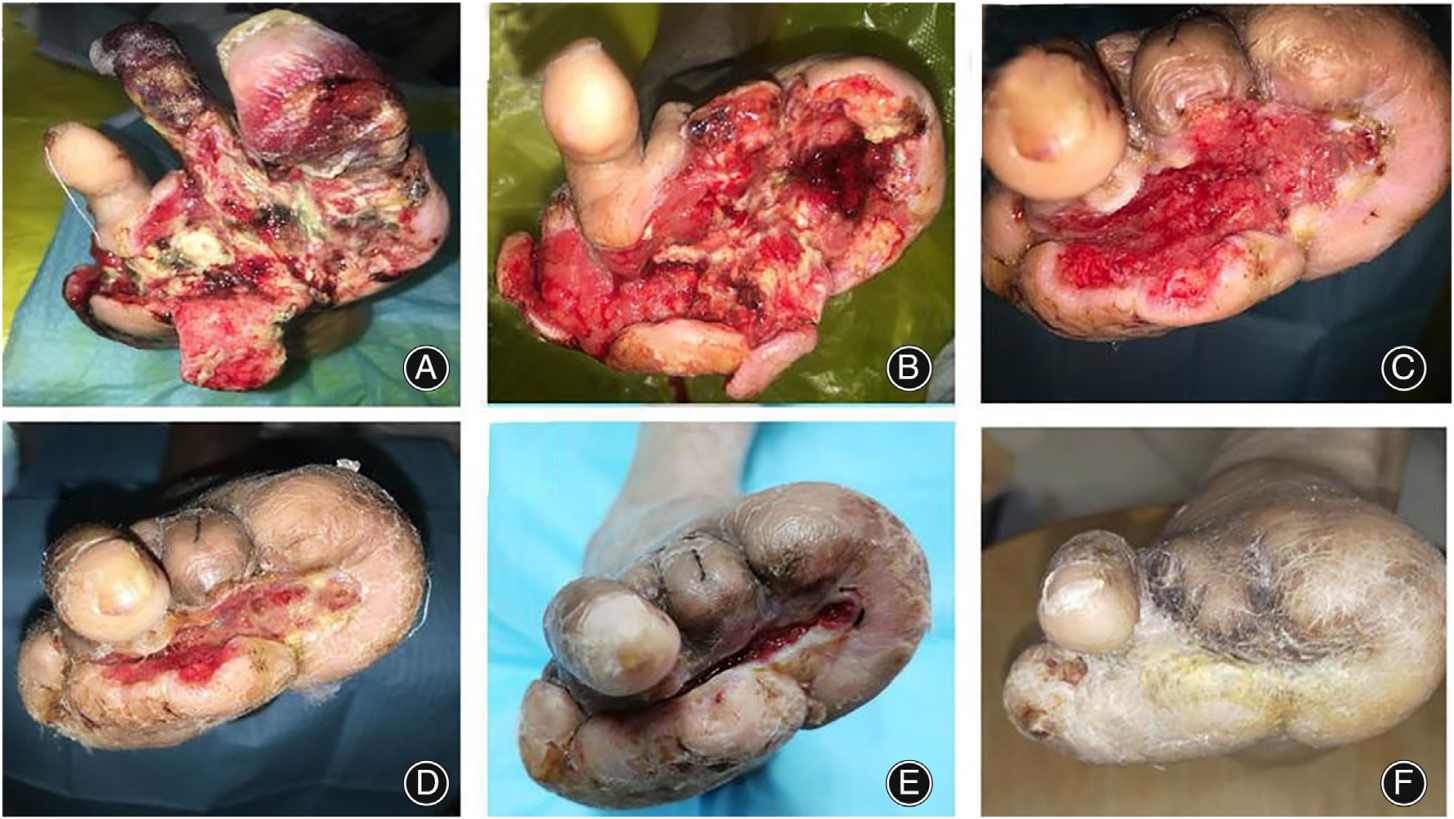
Photograph showing the effects of transverse tibial bone transport in a 50‐year‐old man with severe diabetic foot ulcer. (A) This image shows ulcers before surgery. The foot muscles, tendon, and bone were exposed, and purulent secretion were obvious. The fourth and fifth toes had been amputated and gangrene of the first and second toes was evident. (B) Two weeks postoperatively, the necrotic tissues had been removed during debridement. (C) Six weeks postoperatively, the wound is dry and clean, and the granulation tissue is fresh. (D) Eight weeks postoperatively, the regenerated epithelium gradually grows from the four sides to the center of the wound; (E) Ten weeks postoperatively, the ulcer area is significantly reduced. (F) Twelve weeks postoperatively, the ulcer was completely healed

**Fig. 3:**
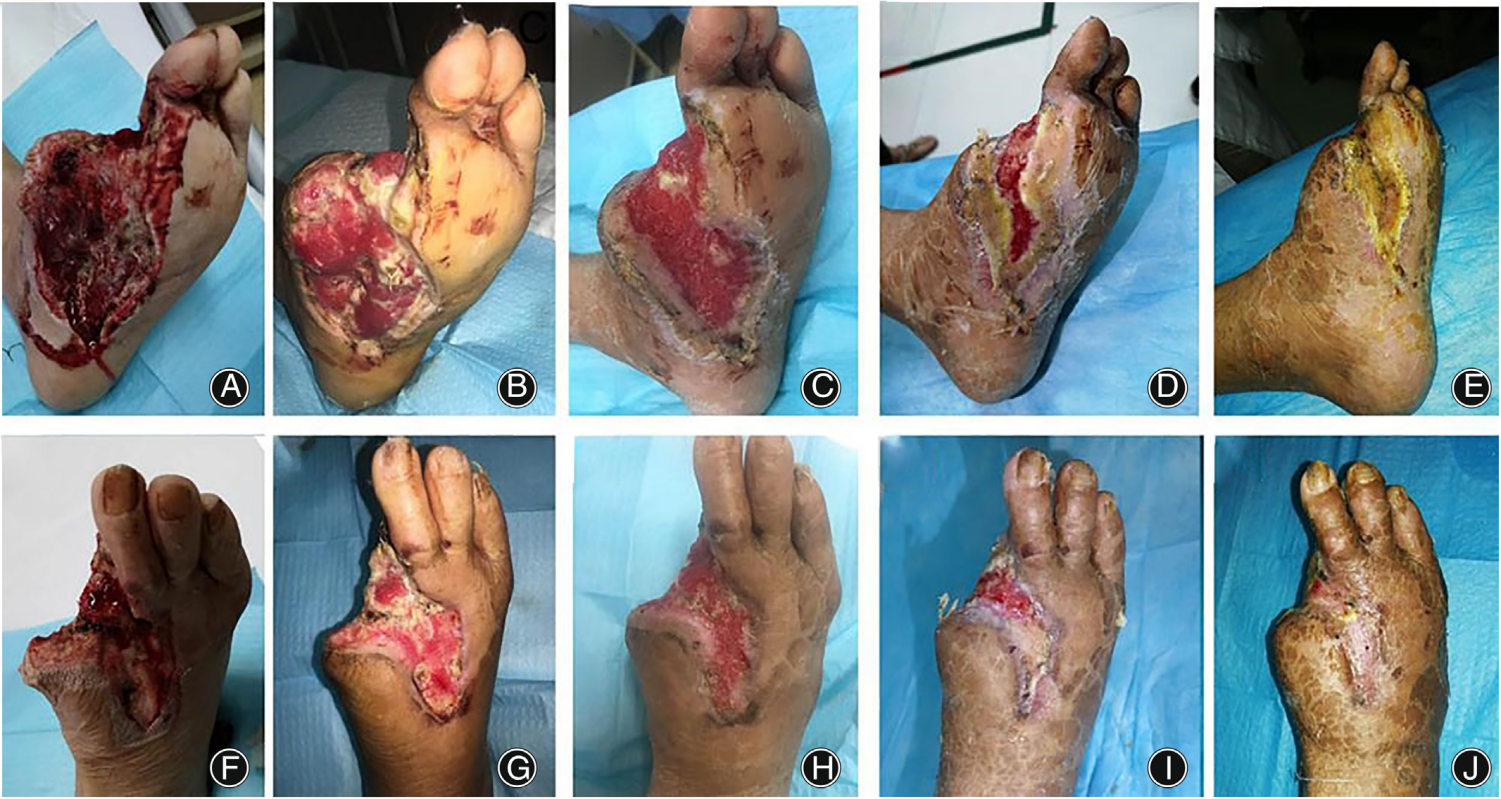
Photograph showing the effects of transverse tibial bone transport in a 55‐year‐old man with severe diabetic foot ulcer. (A–F) This image shows ulcers before surgery. Foot ulcers have huge wounds. The first and second toes had been amputated. (B–G) Four weeks postoperatively, a large amount of granulation tissue grows on the wound. (C–H) Eight weeks postoperatively, epithelialization of the wound surface. (D–I) Twelve weeks postoperatively, epithelialization of the wound surface gradually covers the wound surface. (E–J) Sixteen weeks postoperatively, the ulcer was completely healed

The skin temperature of the foot gradually increased and the pain in the foot gradually improved. In most patients, on the 7th day after transverse transport, red granulation tissue gradually appeared in the ulcers, and the ulcers started to show signs of healing at 21 days (Figures 2 and 3).

### Clinical Assessment

**Fig. 4:**
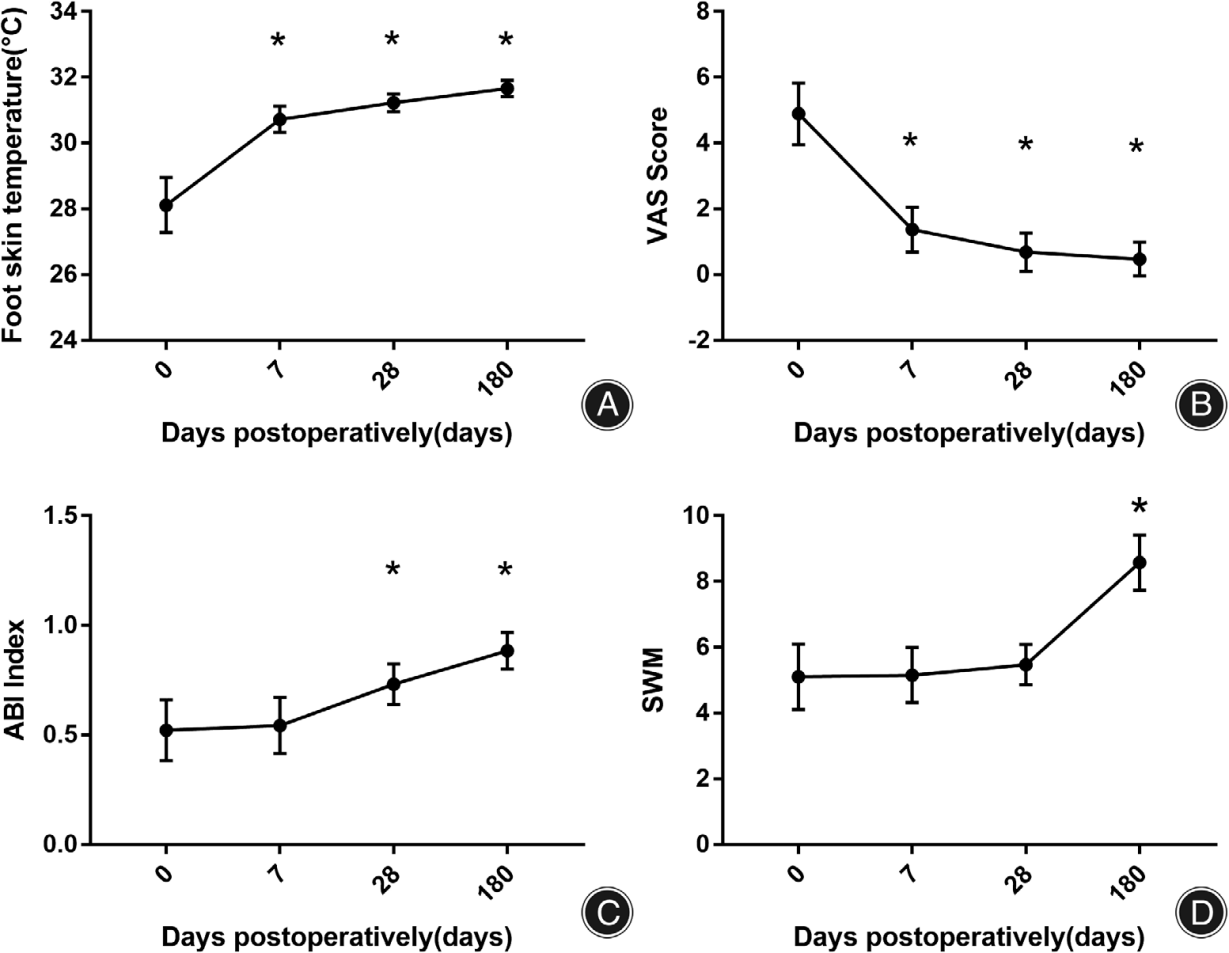
Clinical assessment. (A) Foot skin temperature; (B) VAS score; (C) ABI score; (D) SWM. *p < 0.05 compared to preoperative measurement

The foot skin temperatures on the 7th day after surgery, on the 28th day after surgery, and on the 180th day after surgery were all significantly higher than that before surgery (*p* < 0.05) (Figure 4A). The VAS pain scores on the 7th day after surgery, on the 28th day after surgery, and on the 180th day after surgery were all significantly lower than that before surgery (*p* < 0.05) (Figure 4B). The ABI score on the 28th day after surgery and on the 180th day after surgery were significantly higher than that before surgery (*p* < 0.05) The SWM was significantly decreased on the 180th day after operation (Figure 4C) (Table 3).

**TABLE 3:** The comparison of foot skin temperatures, ABI index, VAS score, and SWM (^−^X ± s)

	Foot skin temperature (°C)	ABI index	VAS score	SWM
Preoperative	28.40 ± 0.84	0.54 ± 0.13	4.97 ± 0.88	5.11 ± 0.92
D7 after surgery,	30.79 ± 0.57*	0.55 ± 0.12	1.42 ± 0.64*	5.21 ± 0.84
D28 after surgery,	31.49 ± 0.45*	0.73 ± 0.08*	0.76 ± 0.54*	5.42 ± 0.68
D180 after surgery,	32.12 ± 0.67*	0.88 ± 0.07*	0.37 ± 0.49*	8.53 ± 0.86*
F value	202.296	116.730	304.632	589.870
*p* value	0.000	0.000	0.000	0.000

Abbreviations: ABI, ankle brachial index; SWM, semmes‐weinsteinmonofilament; VAS, visual analog scale.

### Comparison of Fasting Blood Glucose (FBG) and Hemoglobin A1c (HbAlc)

The preoperative FBG was 7.27 ± 0.52, while it was 6.66 ± 0.34 at 28 days after surgery, which was significantly lower than that before the surgery (*p* < 0.05). The preoperative HbAlc was 7.13 ± 0.57, while it was 6.72 ± 0.36 at 28 days after surgery and 6.57 ± 0.25 at 180 days after surgery, respectively, which were significantly lower than that before the surgery (*p* < 0.05). (Table 4 **).**

**TABLE 4:** Comparison of FBG and HbAlc

	FBG	HbAlc
preoperative	7.27 ± 0.52	7.13 ± 0.57
D7 after surgery	7.23 ± 0.27	7.01 ± 0.45
D28 after surgery	6.66 ± 0.34*	6.72 ± 0.36*
D180 after surgery	6.85 ± 0.42	6.57 ± 0.25*
*F* value	8.322	5.829
*p* value	0.001	0.007

Abbreviations: Date were presented as mean ± standard deviation.

FBG, fasting blood glucose; *p < 0.05 compared to preoperative.

### Imaging of a Typical Case

**Fig. 5:**
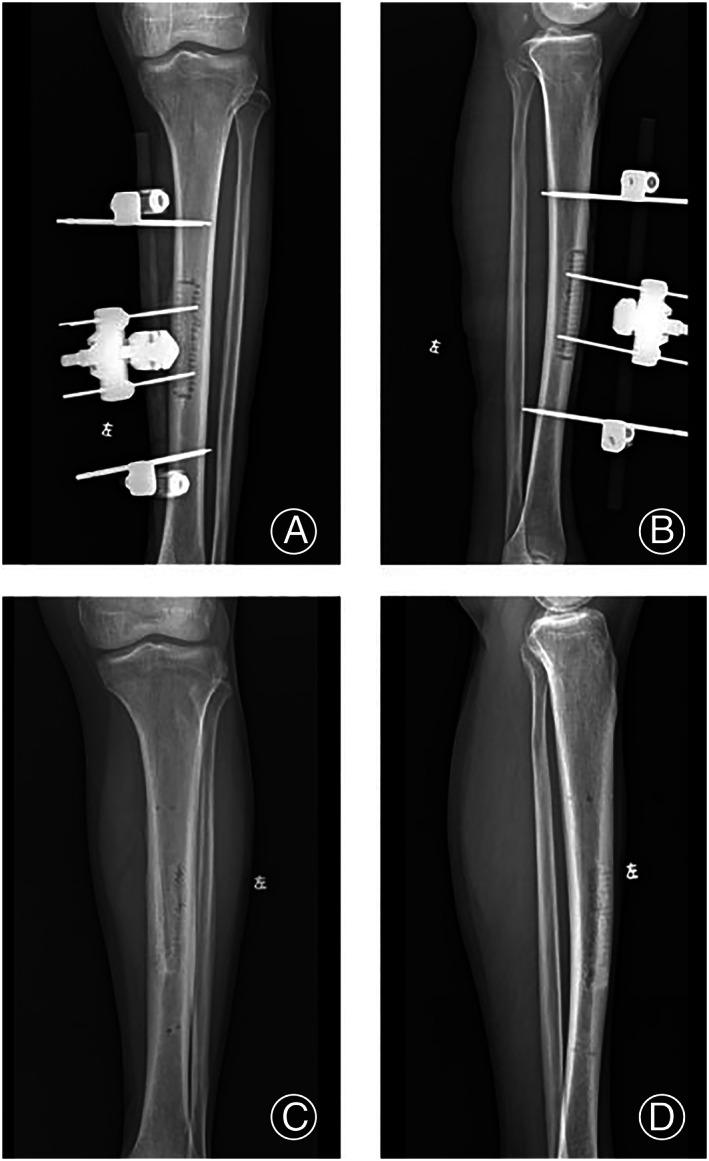
Tibial osteotomy. (A, B) Tibial osteotomy is moved laterally; (C, D) Tibial osteotomy area heals well

An X‐ray film of the osteotomy area on the 1st day after transverse bone transfer showed lateral bone movement (>Figure 5A,B), and bone uplift was observed in X‐ray films after 2 weeks of stretching. X‐ray films at 3 months after surgery showed bone healing after the removal of the external fixator (Figure 5C,D).

**Fig. 6:**
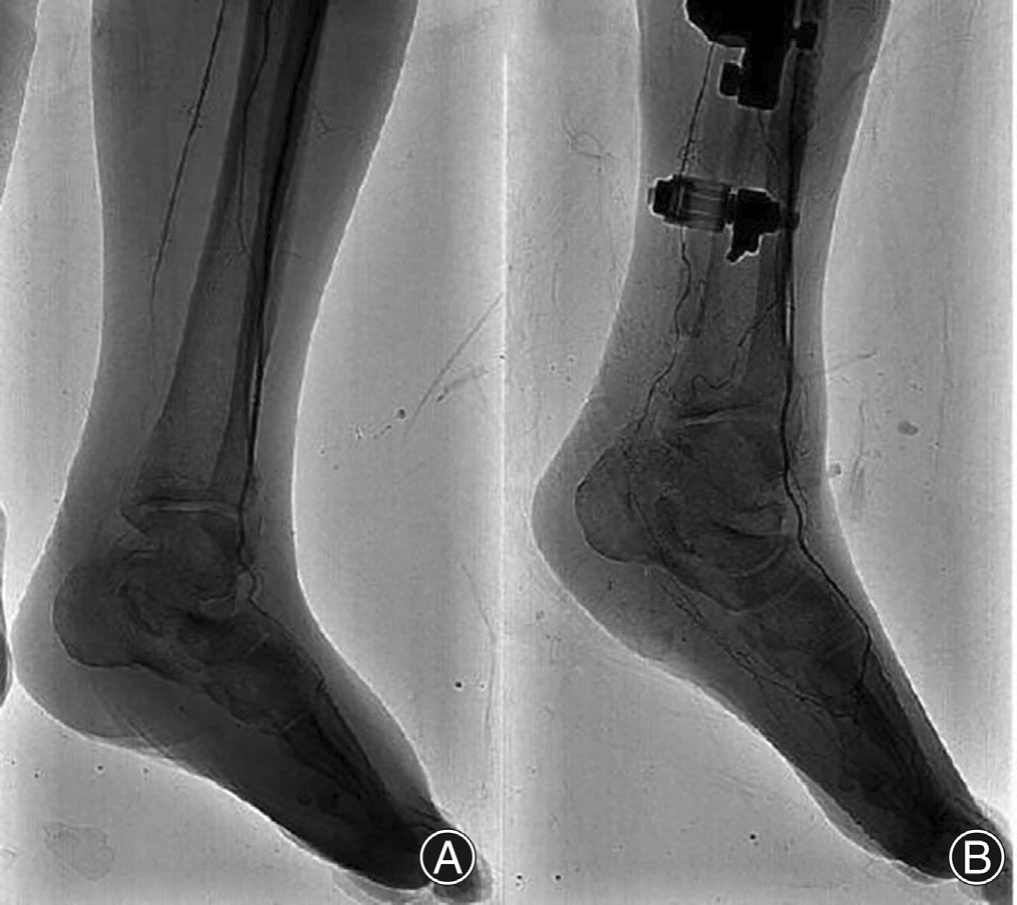
Imaging of a typical case. (A) Preoperative DSA shows blurred development of the calf and foot arteries and surrounding blood vessels. (B) Three months after surgery, DSA showed the thickening of the calf and foot arteries, clear visualization, and a rich vascular network

DSA of the left lower extremity before surgery showed blurred development of the calf and foot arteries and the surrounding vascular network (Figure 6A). DSA of the left lower extremity 3 months after surgery showed the thickening of the calf and foot arteries, clear visualization, and movement around the bone, forming a rich network of blood vessels (Figure 6B).

### The Measurement of VEGF, bFGF, EGF, and PDGF

**Fig. 7:**
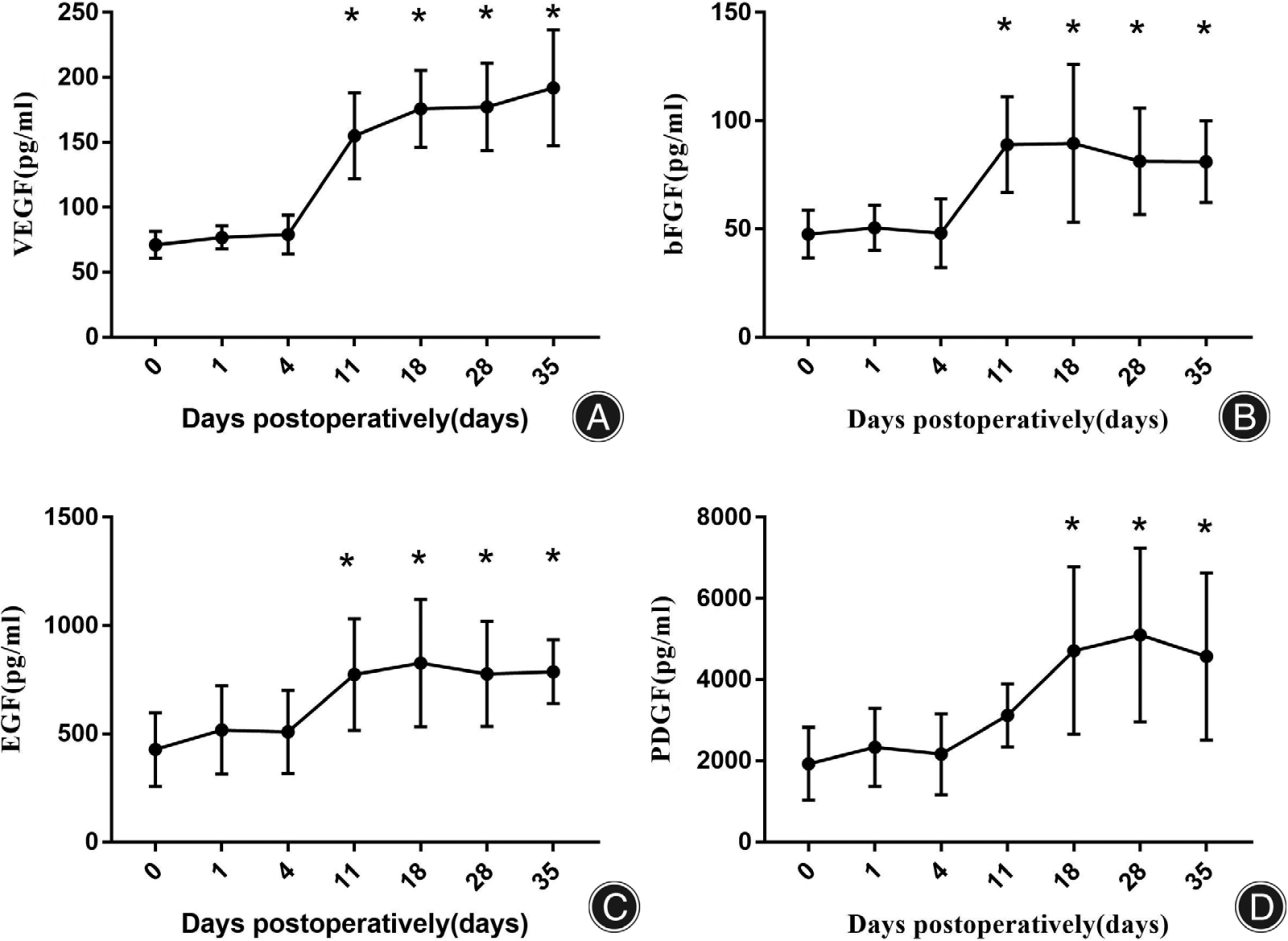
Expression of VEGF, bFGF, EGF, and PDGF. (A) VEGF; (B) bFGF; (C) EGF; (D) PDGF. *p < 0.05 compared to preoperative measurements

The expression of VEGF, bFGF, and PDGF on the 11th day after surgery, on the 18th day after surgery, on the 28th day after surgery, and on the 35th day after surgery was significantly higher than that before surgery (*p* < 0.05). The expression of EGF on the 18th day after surgery, on the 28th day after surgery, and on the 35th day after surgery was significantly higher than that before surgery (*p* < 0.05) (Figure 7) (Table 5).

**TABLE 5:** The expression of VEGF, bFGF, EGF, and PDGF in serum (pg/ml)

	VEGF	bFGF	EGF	PDGF
Preoperativeive	71.19 ± 10.29	47.61 ± 10.91	427.69 ± 169.54	1932.74 ± 897.21
D1 after surgery	76.85 ± 8.91	50.57 ± 10.39	518.36 ± 204.48	2334.86 ± 960.27
D4 after surgery	79.02 ± 15.06	48.11 ± 15.87	509.55 ± 192.58	2168.18 ± 995.05
D11 after surgery	155.01 ± 33.01*	88.99 ± 22.05*	774.11 ± 257.72	3123.13 ± 778.73*

### Complications

No deformity was found in the follow‐up. During the follow‐up period, no ulcers recurred, and no amputation was needed. Superficial surgical wound complications occurred in one (5.26%) patient during the hospitalization, which was treated with dressing. Fortunately, wound healing was achieved without deep wound infection. No other complications were found in these patients, such as movement area infection, nail tract infection, skin flap necrosis, tibial fracture, loosening, or rupture of the external fixator.

## Discussion

In this study, we have observed that transverse tibial bone transport can improve the blood circulation of the affected limbs, promote the healing of diabetic foot wounds, and reduce the amputation rate of the affected limbs. Therefore, transverse tibial bone transport can promote the healing of Wagner Stage 4 diabetic foot.

### Transverse Distraction Technique

In this study, we reconfirmed that the transverse distraction technique proposed by Ilizarov is capable of promoting angiogenesis and is clinically feasible. In patients with diabetic foot, the wound healing rate and limb salvage rate 1 year after surgery were very high, and the clinical curative effect was satisfactory.

The action of repeated mechanical stretching of the transverse tibial bone transport stimulated blood vessel regeneration and improved blood circulation in the leg and foot to promote the healing of the diabetic foot wounds. We observed that the granulation tissue of the foot wound was reddish, soft, and moist in the process of transverse tibial bone transport. The skin around the wound gradually closed to the wound, and the wound was repaired smoothly. A previous study on transverse tibial bone transport found that the regeneration of capillaries was very active, and the number of blood vessels per unit area of the skin on the movement side of the limb increased significantly with the transverse growth of the skin on the removed side^17^. An experimental study of transverse tibial bone transport in canines also showed that there was more capillary regeneration around the removed bone mass^18^. Active regeneration of the micro vascular network was observed in the gap of the distraction region of tibial osteotomy. Tension is used in active tissue to pull the tibial osteotomy at a speed of 1 mm per day, which can regenerate the capillaries well^14^. Ohashi *et al*.^19^ reported that distraction osteogenesis activated angiogenesis and maintained increasing vascularity, and new vessels persisting for a certain time after distraction. Choi *et al*.^20^ believed that distraction osteogenesis machinery stimulated the development of all related soft tissues and enhanced the blood supply by activating angiogenesis.

The clinical effect of this technique should depend on the persistence of the regenerative blood vessels over a period of time after distraction osteogenesis^21^. DSA examination of the affected limb after surgery showed the large calf arteries and the foot arteries of the affected limb. It is considered that lateral tibial bone transport can improve the microcirculation of the leg and foot.

### Improvement of VAS and ABI

The postoperative VAS score was significantly decreased on the 7th day, 28th day, and 180th day after operation compared with that before the operation. The skin temperature of the foot was significantly increased on the 7th day, 28th day, and 180th day after the operation. The ABI score was significantly increased on the 28th day and 180th day after the operation. The improvement of VAS and ABI might indicate the improvement of neuropathy, therefore we have further evaluated the neuropathy by Semmes‐Weinstein monofilament test. The Semmes‐Weinstein monofilament test was significantly decreased on the 180th day after operation. These results suggested that the skin temperature and pain of the affected foot can be obviously improved with this procedure. A previous study has evaluated autologous bone marrow mononuclear cell and VEGF165 gene therapy in patients with diabetes mellitus suffering from critical limb ischaemia, and found that the ABI improved significantly 3 months after therapy, while VAS decreased after 3 months, which was in accordance with our results^22^.

### The Size of Osteotomy and the Length of Bone Removal

There is still a lack of evidence defining the size of osteotomy and the length of bone removal. In our experience, the size of the osteotomy was 7 cm × 1.8 cm, and good clinical effects were obtained. It may be that slow stimulation of biomechanics is the most important factor. The length of bone removal after the operation was determined according to the accordion technique, and the removal time was 4 weeks. Mechanical stimulation could promote tissue regeneration, and at the same time, the tibial osteotomy mass could be reduced.

Traction which is too slow would lead to premature calcification of new bone, which cannot be prolonged, but traction which is too fast would lead to a lack of blood supply in the tissue and local tissue necrosis and not be conducive to the formation of blood vessels. Ilizarov suggested that the reasonable speed and frequency of traction were 0.5 mm/day and 2 times/day, which could promote vascular regeneration of the affected limbs^13^. Traction speed can be adjusted at any time according to clinical needs. Especially when the osteotomy block is pulled outward, if the pull were too fast, the tension of the skin around the osteotomy area will be too large, which may lead to ischemic necrosis. Once skin necrosis occurs, it may lead to the failure of the operation and even the risk of amputation. The velocity of bone movement should be adjusted according to the skin tension and blood supply around the osteotomy area. If the blood supply of the skin is poor, the osteotomy block can be pressed back.

### Expression of Angiogenesis

Distraction osteogenesis can stimulate the production of angiogenesis factors, thereby promoting the high expression of angiogenesis, VEGF, and bFGF in both new bone and new muscle^13^. Distraction osteogenesis can cause systemic reactions in the body. This promotes the release of a large number of inflammatory mediators, hormones, stem cells, and growth factors^23^, ^24^. Distraction osteogenesis not only promotes the local angiogenesis of new bone but also stimulates the high expression of angiogenic factors and their receptors in the skeletal system throughout the body^15^.

VEGF is an indispensable cytokine in the process of wound healing and is involved in all stages of wound healing, while local application of VEGF can vascularize the wound and enhance the healing ability of the wound^25^, ^26^, ^27^, ^28^. bFGF plays an important role in wound repair by promoting fibroblast proliferation, collagen proliferation, and endothelial vascularization^29^, ^30^. EGF promotes the division of various tissue cells and participates in the regulation of cell proliferation, migration, and differentiation^31^. PDGF can stimulate cell proliferation, accelerate granulation tissue formation, and promote wound healing^32^. The results of this study showed that on the 11th, 18th, 28th, and 35th days after transverse transport, the expression of VEGF, bFGF, EGF, and PDGF in serum was increased compared with that before operation. These results suggest that transverse tibial bone transport can promote the expression of VEGF, bFGF, EGF, and PDGF through the process of tibial osteotomy and further promote angiogenesis. To rebuild and restore blood flow, neovascularization can provide nutrients for repairing damaged tissue and promote the healing of diabetic foot wounds.

### Limitations

There are several limitations in this study. First, this is an observational trial with small sample size and single arm design, which makes it difficult to draw a direct conclusion that transverse tibial bone transport is better for the treatment of diabetic foot. Second, multiple surgeons participated in the registration of patients based on the inclusion and exclusion criteria of our group. However, all procedures were performed by a senior orthopaedic surgeon using the same surgical technique, which eliminated the effect of multiple surgeons with different levels of expertise in transverse tibial bone transport for diabetic foot. Third, the follow‐up period was short, and the number of patients is limited. Further multicenter study to observe the long‐term curative effect is still needed. However, the authors believe that it is very important to illustrate our early experiences and complications in this procedure because there are a limited number of studies concerning whether transverse tibial bone transport is better for diabetic foot. Finally, the wound healing was not quantitatively evaluated.

### Conclusion

In conclusion, transverse tibial bone transport is a reasonable and effective surgical method worthy of clinical popularization. It can improve the blood circulation of the affected limbs, promote the healing of diabetic foot wounds, and reduce the amputation rate of the affected limbs. In the treatment of diabetic foot, transverse tibial bone transport can significantly increase the expression of serum angiogenic factors in the early stage, which may be the mechanism of promoting the healing of diabetic foot wounds. Further study with control group and large sample size is needed to further prove our conclusion.

## Fundings

This work was supported by the Fund of Guangdong Second People's Hospital (YQ2019‐009, 3D‐A2020002); National Natural Science Foundation of China (81972083); Natural Science Foundation of Guangdong Province (2017A030313736); Guangzhou Science and Technology Plan Project (201804010226).

## Authorship

Yong Qi was the guarantor of integrity of the entire study; Hongtao Sun carried out the study concepts; Wenjun Li carried out the study design; Ya Chen performed the definition of intellectual content; Hanyu Lu was dedicated to the literature research; Changpeng Xu was involved in the clinical studies; Shuanji Ou carried out the experimental studies, data acquisition, data analysis, and manuscript editing; Yang Yang performed the statistical analysis; Guitao Li handled the manuscript review. All authors have read and approved this article.

## Competing interests

The authors declare no conflicts of interest.
